# The Natural History and Transmission Potential of Asymptomatic Severe Acute Respiratory Syndrome Coronavirus 2 Infection

**DOI:** 10.1093/cid/ciaa711

**Published:** 2020-06-04

**Authors:** Nguyen Van Vinh Chau, Vo Thanh Lam, Nguyen Thanh Dung, Lam Minh Yen, Ngo Ngoc Quang Minh, Le Manh Hung, Nghiem My Ngoc, Nguyen Tri Dung, Dinh Nguyen Huy Man, Lam Anh Nguyet, Le Thanh Hoang Nhat, Le Nguyen Truc Nhu, Nguyen Thi Han Ny, Nguyen Thi Thu Hong, Evelyne Kestelyn, Nguyen Thi Phuong Dung, Tran Chanh Xuan, Tran Tinh Hien, Nguyen Thanh Phong, Tran Nguyen Hoang Tu, Ronald B Geskus, Tran Tan Thanh, Nguyen Thanh Truong, Nguyen Tan Binh, Tang Chi Thuong, Guy Thwaites, Le Van Tan, Oxford University Clinical Research Unit COVID-19 Research Group, Nguyen Van Vinh Chau, Nguyen Thanh Dung, Le Manh Hung, Huynh Thi Loan, Nguyen Thanh Truong, Nguyen Thanh Phong, Dinh Nguyen Huy Man, Nguyen Van Hao, Duong Bich Thuy, Nghiem My Ngoc, Nguyen Phu Huong Lan, Pham Thi Ngoc Thoa, Tran Nguyen Phuong Thao, Tran Thi Lan Phuong, Le Thi Tam Uyen, Tran Thi Thanh Tam, Bui Thi Ton That, Huynh Kim Nhung, Ngo Tan Tai, Tran Nguyen Hoang Tu, Vo Trong Vuong, Dinh Thi Bich Ty, Le Thi Dung, Thai Lam Uyen, Nguyen Thi My Tien, Ho Thi Thu Thao, Nguyen Ngoc Thao, Huynh Ngoc Thien Vuong, Pham Ngoc Phuong Thao, Phan Minh Phuong, Dong Thi Hoai Tam, Evelyne Kestelyn, Donovan Joseph, Ronald Geskus, Guy Thwaites, H Rogier van Doorn, Ho Van Hien, Huynh Le Anh Huy, Huynh Ngan Ha, Huynh Xuan Yen, Jennifer Van Nuil, Jeremy Day, Joseph Donovan, Katrina Lawson, Lam Anh Nguyet, Lam Minh Yen, Le Nguyen Truc Nhu, Le Thanh Hoang Nhat, Le Van Tan, Sonia Lewycka Odette, Louise Thwaites, Maia Rabaa, Marc Choisy, Mary Chambers, Motiur Rahman, Ngo Thi Hoa, Nguyen Thanh Thuy Nhien, Nguyen Thi Han Ny, Nguyen Thi Kim Tuyen, Nguyen Thi Phuong Dung, Nguyen Thi Thu Hong, Nguyen Xuan Truong, Phan Nguyen Quoc Khanh, Phung Le Kim Yen, Sophie Yacoub, Thomas Kesteman, Nguyen Thuy Thuong Thuong, Tran Tan Thanh, Tran Tinh Hien, Vu Thi Ty Hang, Nguyen Tri Dung, Le Hong Nga

**Affiliations:** 1 Hospital for Tropical Diseases, Ho Chi Minh City, Vietnam; 2 Oxford University Clinical Research Unit, Ho Chi Minh City, Vietnam; 3 Children’s Hospital 1, Ho Chi Minh City, Vietnam; 4 Center for Disease Control and Prevention, Ho Chi Minh City, Vietnam; 5 Centre for Tropical Medicine and Global Health, Nuffield Department of Medicine, University of Oxford, Oxford, United Kingdom; 6 Cu Chi Hospital, Ho Chi Minh City, Vietnam; 7 Department of Health, Ho Chi Minh City, Vietnam

**Keywords:** COVID-19, SARS-CoV-2, coronaviruses, pandemic, Vietnam

## Abstract

**Background:**

Little is known about the natural history of asymptomatic severe acute respiratory syndrome coronavirus 2 (SARS-CoV-2) infection.

**Methods:**

We conducted a prospective study at a quarantine center for coronavirus disease 2019 in Ho Chi Minh City, Vietnam. We enrolled quarantined people with reverse-transcription polymerase chain reaction (RT-PCR)–confirmed SARS-CoV-2 infection, collecting clinical data, travel and contact history, and saliva at enrollment and daily nasopharyngeal/throat swabs (NTSs) for RT-PCR testing. We compared the natural history and transmission potential of asymptomatic and symptomatic individuals.

**Results:**

Between 10 March and 4 April 2020, 14 000 quarantined people were tested for SARS-CoV-2; 49 were positive. Of these, 30 participated in the study: 13 (43%) never had symptoms and 17 (57%) were symptomatic. Seventeen (57%) participants imported cases. Compared with symptomatic individuals, asymptomatic people were less likely to have detectable SARS-CoV-2 in NTS collected at enrollment (8/13 [62%] vs 17/17 [100%]; *P* = .02). SARS-CoV-2 RNA was detected in 20 of 27 (74%) available saliva samples (7 of 11 [64%] in the asymptomatic group and 13 of 16 [81%] in the symptomatic group; *P* = .56). Analysis of RT-PCR positivity probability showed that asymptomatic participants had faster viral clearance than symptomatic participants (*P* < .001 for difference over the first 19 days). This difference was most pronounced during the first week of follow-up. Two of the asymptomatic individuals appeared to transmit SARS-CoV-2 to 4 contacts.

**Conclusions:**

Asymptomatic SARS-CoV-2 infection is common and can be detected by analysis of saliva or NTSs. The NTS viral loads fall faster in asymptomatic individuals, but these individuals appear able to transmit the virus to others.

## Teaser

Forty-three percent (13/30) of confirmed SARS-CoV-2–infected individuals were asymptomatic, with the virus detected in both saliva and nasopharyngeal/throat swabs. Viral clearance was faster in asymptomatic individuals, but they still appeared able to pass the infection to others.

The rapid global spread of severe acute respiratory syndrome coronavirus 2 (SARS-CoV-2) has prompted the World Health Organization (WHO) to declare a pandemic. As of 23 April 2020, > 2.6 milllion confirmed cases and > 180 000 deaths have been reported globally. Vietnam reported its first confirmed cases on 22 January 2020 [1]. Yet, as of 24 April 2020, a total of 270 cases have been reported, with no deaths [2].

The clinical syndrome caused by SARS-CoV-2 is called coronavirus disease 2019 (COVID-19) [3], an infectious disease that varies from mild to severe, life-threatening respiratory infection. Asymptomatic infection with SARS-CoV-2 has been reported [4–6] in up to 43% of those with proven infection in a recent Italian study [7]. SARS-CoV-2–infected patients can be infectious prior to symptom (COVID-19) development and cause transmission [8, 9]. Furthermore, there is some evidence demonstrating the transmisson potential of those with reverse-transcription polymerase chain reaction (RT-PCR)–confirmed infection who never develop symptoms during their infection (asymptomatic transmission) [4, 5, 7], suggesting that asymptomatic infection may play an important role in the spread of SARS-CoV-2.

SARS-CoV-2 is transmitted by respiratory droplets from infected people if they cough and/or sneeze. In the absence of respiratory symptoms, the mechanism by which asymptomatic individuals transmit SARS-CoV-2 to their contacts remains unclear. In most countries, only patients with moderate or severe disease are admitted to hospital for management [10–13], leaving those without symptoms, or with mild disease, uncharacterized, especially concerning their laboratory and virological findings.

We therefore studied asymptomatic individuals with SARS-CoV-2 infection and those with mild disease identified as part of ongoing contact tracing and airport quarantine implemented in Ho Chi Minh City (HCMC), Vietnam. Our aims were to compare the duration of viral detection and abundance in the respiratory tract, including saliva, of asymptomatic and mildly symptomatic patients, and to assess their ability to transmit the virus to others.

## MATERIALS AND METHODS

### Vietnam Containment Approach

**Figure 1.**
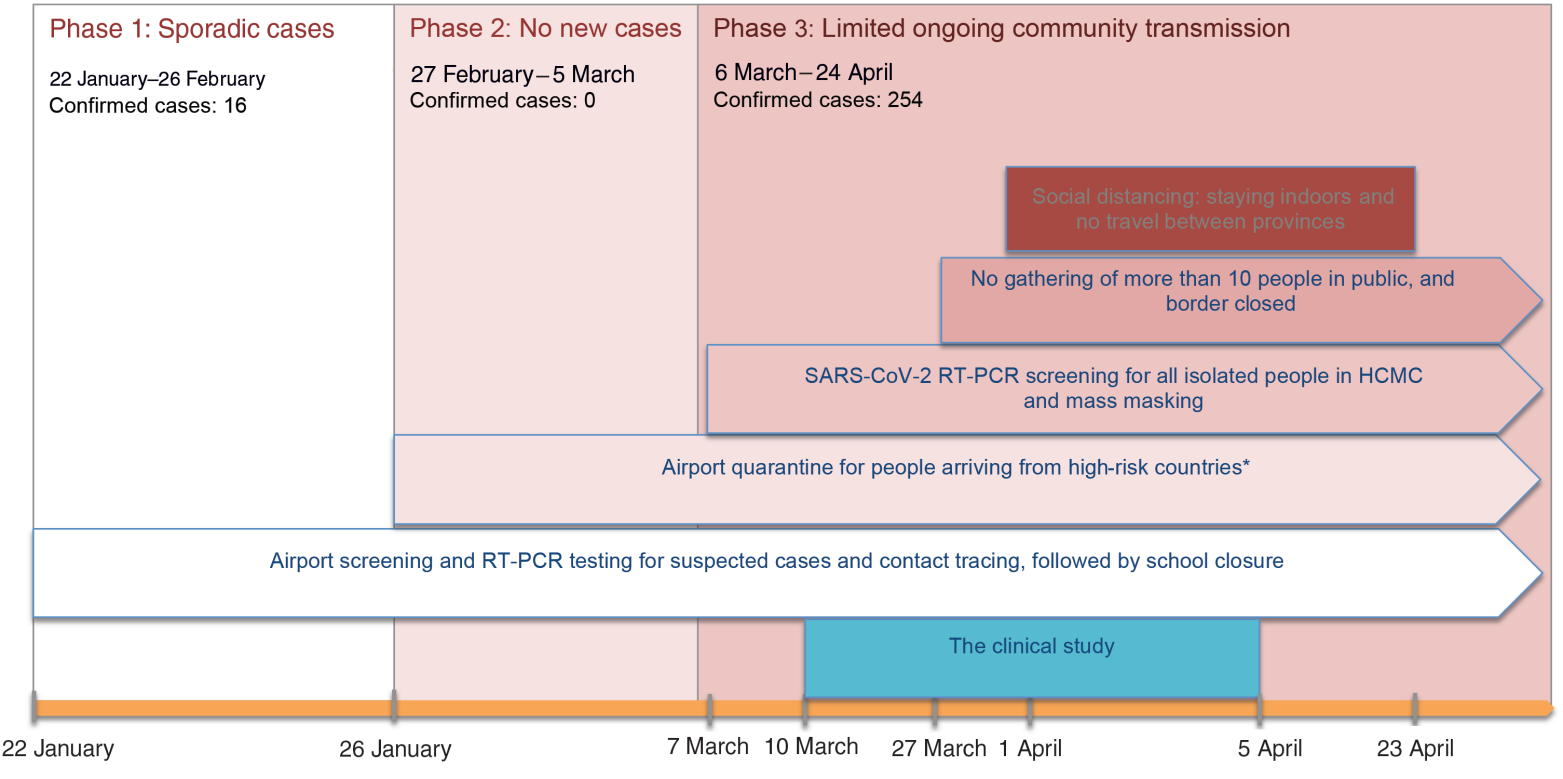
Timelines of containment strategies applied in Vietnam and in Ho Chi Minh City (HCMC) since the beginning of 2020 as the epidemic/pandemic progresses alongside the implementation of severe acute respiratory syndrome coronavirus 2 (SARS-CoV-2) reverse-transcription polymerase chain reaction (RT-PCR) testing and the duration of the clinical study. *Initially China, followed by Korea, other European counties (Italy, France, United Kingdom, etc.) and the United States.

Since January 2020, various control measures, including isolation of confirmed cases, contact tracing, airport quarantine, and social distancing have been implemented in Vietnam with increasing stringency as the pandemic progressed worldwide (Figure 1) [14, 15]. Accordingly, anyone known to have been in contact with a confirmed COVID-19 case, or having traveled to Vietnam from a COVID-19–affected country, were isolated for ≥ 14 days at a designated isolation center.

From the second week of March 2020, all isolated individuals were subject to serial SARS-CoV-2 nasopharyngeal/throat swab (NTS) screening by real-time RT-PCR. A confirmed case was established if 2 independent RT-PCR assays (E gene and RNA-dependent RNA-polymerase [RdRp] RT-PCR assays) were positive [16]. Confirmed cases were admitted to a designated COVID-19 hospital for follow-up until they recovered and/or had at least 2 consecutive days with negative SARS-CoV-2 RT-PCR NTSs [17].

### Setting

The Hospital for Tropical Diseases (HTD) is a tertiary referral infectious diseases hospital responsible for receiving and treating patients with COVID-19 in southern Vietnam. From January 2020 to the first week of April, HTD was responsible for RT-PCR screening of 80% of quarantined people in HCMC.

**Figure 2.**
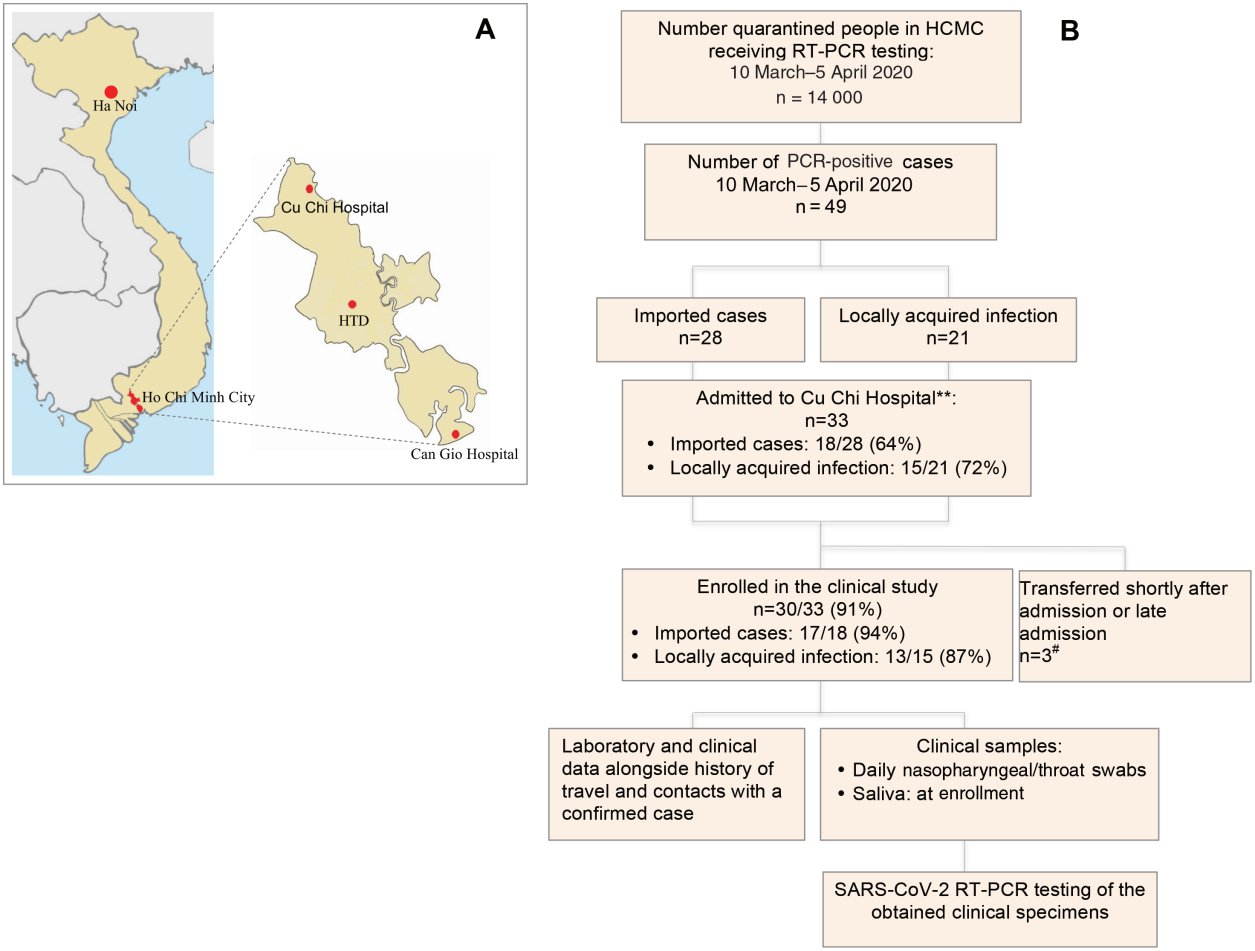
Settings and the clinical study. *A*, Map showing the location of the Hospital for Tropical Diseases (HTD) main campus and its designated coronavirus disease 2019 centers in Cu Chi, where the clinical study was conducted, and Can Gio. *B*, Flowchart illustrating the results of reverse-transcription polymerase chain reaction screening of quarantined people between 10 March and 5 April 2020, and the enrollment of patients in the clinical study. *Extrapolated from data extracted from the HTD database system. During this period, HTD tested a total of 11 052 cases, accounting for 80% of isolated people in Ho Chi Minh City (HCMC). **The remaining cases were either treated at the main campus of HTD in HCMC or at the other designated isolation center (Can Gio Hospital). ^#^One was transferred to the main campus of HTD 10 hours after admission and 2 were transferred from the main campus of HTD after 5 and 6 days of hospitalization and were not enrolled because of enrollment competition. Maps were obtained from https://mapchart.net/. Abbreviations: HCMC, Ho Chi Minh City; HTD, Hospital for Tropical Diseases; PCR, polymerase chain reaction; RT-PCR, reverse-transcription polymerase chain reaction; SARS-CoV-2, severe acute respiratory syndrome coronavirus 2.

In addition to its main campus in the center of HCMC, HTD has 2 designated 300-bed centers for the care of confirmed/suspected cases with COVID-19, namely Cu Chi and Can Gio Hospitals, located approximately 60 km to the west and east, respectively, of HCMC (Figure 2A). The present study was conducted at Cu Chi Hospital.

### Patient Enrollment and Data Collection

We enrolled individuals with confirmed SARS-CoV-2 infection admitted to Cu Chi Hospital from 10 March to 4 April 2020. From each participant, we prospectively collected demographic and clinical data, travel history, and information concerning contact with confirmed COVID-19 cases, using standardized paper case record forms.

We collected NTSs, combining them into a single tube containing 1 mL of viral transport medium. NTSs were taken daily from enrollment to hospital discharge (Figure 2B). Additionally, a saliva sample was obtained at enrollment. After collections, clinical samples were stored at 4°C at the study site and were then transferred to the HTD laboratory in HCMC within 4 hours for analysis.

### Viral RNA Extraction and SARS-CoV-2 RT-PCR Analysis

We manually extracted viral RNA from 140 μL of NTS and saliva samples (if volume was sufficient for testing) using the QIAamp viral RNA kit (QIAgen GmbH, Hilden, Germany), and then recovered the cleaned-up RNA in 50 μL of elution buffer provided with the kit. Since we enrolled patients who had a confirmed diagnosis by 2 independent RT-PCRs (Egene and RdRp assays) as per the WHO recomendation [16], we used E gene assay for testing of samples collected from enrollment onward. Real time RT-PCR was carried out as previously described [16].

### Data Analysis

For viral load–associated analysis, in the absence of quantitative RT-PCR results, we use cycle threshold (Ct) values as surrogates. We used the *t* test to compare the difference in measured Ct values obtained at enrollment between the 2 groups, Wilcoxon signed-rank test to compare the measured Ct values between NTSs and saliva, and χ ^2^ test to compare 2 proportions. We compared the trend in the detection probability of SARS-CoV-2 and the viral RNA load in NTSs between asymptomatic and symptomatic individuals. For the detection probability, we fitted a logistic regression model that quantifies the probability to test positive over time. We used generalized estimating equations (geepack package in R [18]) to correct for the repeated measurements per individual. We assumed that those who left the study earlier, after several days with a negative test result, remained negative until day 19. For the trend in Ct, we used a zero-inflated mixed-effects model for semicontinuous data (GLMMadaptive package in R). Since the Ct data have an upper threshold of 40.5, we used the transformation Y = 40.5-Ct. Hence, an undetectable viral load was given the value zero. We report the mean value on the original scale, which is a weighted combination of the value 40.5 for those who test negative and the measured value of those who test positive. Additionally, we compared the measured Ct values between the 2 groups; for this we used a random-effects model. Further details can be found in the Supplementary Data. Note that inference with respect to measured Ct values should be interpreted with caution, because the subgroup of negative values is selectively excluded. Apart from R, we also performed the analysis in SPSS version 23.0 (IBM SPSS, Armonk, New York) and generated the figures using GraphPad Prism version 5.04 (GraphPad Software, San Diego, California), and R software.

### Ethical Considerations

This clinical study received approvals from the Institutional Review Board of the HTD and the Oxford Tropical Research Ethics Committee of the University of Oxford. Study participants gave their written informed consent.

## RESULTS

### RT-PCR Screening of Quarantined People

Between 10 March and 4 April 2020, approximately 14 000 people were referred to 1 of 9 designated quarantine centers deployed across HCMC, and were screened for SARS-CoV-2 by RT-PCR of NTSs. Forty-nine people had a positive test, accounting for 96% (49/51) of all reported cases in HCMC during the same period. The other 2 self-presented to local hospitals after falling ill. Of these 49, 33 (67%) were admitted to Cu Chi Hospital, and 30 (30/33 [91%]) agreed to participate in the clinical study (Figure 2B).

### Baseline Characteristics of the Study Participants

Of the 30 study participants, 16 were imported cases (ie, they acquired the infection outside of Vietnam; Supplementary Figure 1), and 14 acquired the infection locally; all 14 had an epidemiological link with 2 community transmission clusters occurring in HCMC during the study period. Of the locally acquired infections, 7 (50%) were asymptomatic, whereas 6 (38%) of the imported cases were asymptomatic. Those with locally acquired infection were more likely to be male than those with infection acquired outside of Vietnam (Table 1).

**Table 1.** Demographics of Imported Cases and Cases of Locally Acquired Infection

Characteristic	Locally Acquired Infections (n = 14)	Imported Cases^a^ (n = 16)
Female sex, No. (%)	4 (29)	11 (69)
Age, y, median (range)	30.5 (23–51)	21.5 (16–60)
Asymptomatic, No. (%)	7 (50)	6 (38)

^a^Defined as cases arriving in Vietnam from abroad and positive for severe acute respiratory syndrome coronavirus 2 as part of airport quarantine and reverse-transcription polymerase chain reaction screening.

Seventeen of the participants had mild respiratory disease (ie, no requirement for supplementary oxygen during hospitalization). None of the participants developed severe disease or progressed from asymptomatic to symptomatic. The other 13 patients had no symptoms or signs of infection throughout their hospital admission. The demographic and laboratory characteristics of the 2 groups were similar at enrollment (Tables 1 and 2). A small proportion of symptomatic patients presented with diarrhea and/or lost their sense of smell. None of the 30 participants had abnormal findings on chest radiographs.

**Table 2.** Baseline Characteristics of the Study Participants

Characteristic	All (N = 30)	Symptomatic (n = 17)	Asymptomatic (n = 13)
Age, y, median (range)	29 (16–60)	27 (18–58)	30 (16–60)
Sex (female/male), No./No.	15/15	9/8	6/7
Arriving in Vietnam from abroad, No. (%)	16 (53)	10 (59)	6 (47)
Locally acquired infection, No. (%)	14 (47)	7 (41)	7 (53)
Nationality, No. (%)			
Vietnamese	19 (63)	12 (71)	7 (53)
Others	11 (37)^a^	5 (29)^b^	6 (46)^c^
Days from confirmed diagnosis to enrollment, median (range)	2 (2–5)	2 (0–3)	2 (1–5)
Days from admission to enrollment, median (range)	1 (0–2)	1 (0–2)	1 (0–2)
Duration of stay, d	16 (9–26)	16 (11–26)	15 (9–23)
Laboratory results^d^, median (range)/normal range			
WBC count , ×10^3^ per μL	5.16 (3.1–9.9)/(4–11)	5.0 (3.4–8.3)	5.51 (3.15–4.83)
Lymphocyte counts, ×10^3^ per μL	1.65 (0.56–2.94)/(1.5–4)	1.47 (0.56–2.94)	1.88 (1.17–2.5)
Hemoglobin, g/dL	14.3 (10–17.3)/(13–18)	14.4 (11.6–16.8)	14.15 (10–17.3)
Hematocrit, %	35.5 (28.5–42.3)/(37–52)	36.5 (28.5–42.3)	36 (35.78–42.27)
Platelet count/μL	257 (130–414)/(150–450)	249 (130–414)	265.5 (174–321)
Glucose, mmol/L	85 (6–340)^e^/(70–130)	84.2 (68–340)^f^	101.65 (64–146)^g^
Creatinine, mg/dL	1.0 (0.9–1.5)^h^/(0.5–1.2)	1.0 (0.9–12.4)^i^	1 (0.96–1.54)^g^
AST, U/L	22.5 (15.4–56.8)^e^/(< 40)	22.5 (17.4–56.8)^i^	17.4 (15.4–32.4)^g^
ALT, U/L	22.3 (9.7–44.9)/(< 37)	24 (10.2–34.8)^i^	19.15 (9.7–44.9)^g^
Clinical signs/symptoms^j^, No. (%)			
Fever	8 (27)	8 (47)	NA
Cough	10 (33)	10 (59)	NA
Rhinorrhea	3 (10)	3 (18)	NA
Fatigue	1 (3)	1 (6)	NA
Diarrhea	3 (10)	3 (18)	NA
Sore throat	6 (20)	6 (36)	NA
Muscle pain	3 (10)	3 (18)	NA
Headache	2 (7)	2 (12)	NA
Abdominal pain	1 (3)	1 (6)	NA
Lost sense of smell	3 (10)	3 (18)	NA
Comorbidity	2 (7)	2 (12)^k^	0

Abbreviations: ALT, alanine aminotransferase; AST, aspartate aminotransferase; NA, not applicable; WBC, white blood cell.

^a^Brazil (n = 3), United Kingdom (UK) (n = 3), Canada (n = 2), United States (n = 1), Czech Republic (n = 1), Italy (n = 1).

^b^UK (n = 2), Canada (n = 1), United States (n = 1), Czech Republic (n = 1).

^c^Brazil (n = 3), UK (n = 1), Canada (n = 1), Italy (n = 1).

^d^One participant had blood collection on day 2; 2 participants on day 3; and 1 participant on day 4 after admission.

^e^n = 17.

^**f**^n = 10.

^g^n = 6.

^h^n = 18.

^i^n = 11.

^j^Others (nausea, vomiting, short breathing, bleeding, and taste disorder) were also recorded, but none presented with these signs/symptoms.

^k^Degenerative spine in 1 and diabetes in 1.

### Viral Detection in NTS and Saliva Samples

Compared with symptomatic patients, those with asymptomatic infection were less likely to have detectable SARS-CoV-2 in NTS samples collected at enrollment (8/13 [62%] vs 17/17 [100%]; *P* = .02). However, 4 of 5 patients who had a negative NTS collected at enrollment had a positive NTS result in 1 of the subsequent sampling days, but with a high Ct value (Supplementary Figure 2), suggesting that these patients had low viral load in their respiratory samples.

Of the 30 study participants, 27 (90%) had a saliva sample collected at enrollment with sufficient volume for RT-PCR analysis. SARS-CoV-2 RNA was detected in 20 of 27 (74%) available saliva samples: 7 of 11 (64%) in the asymptomatic group and 13 of 16 (81%) in the symptomatic group (*P* = .56). There was 1 patient with a negative NTS collected at enrollment but a positive saliva result. Accordingly, a combination of both NTS and saliva samples collected at enrollment slightly increased the diagnostic yield of samples collected at enrollment of the asymptomatic group.

### Quantification of Viral RNA in NTS and Saliva Samples at Enrollment

**Figure 3.**
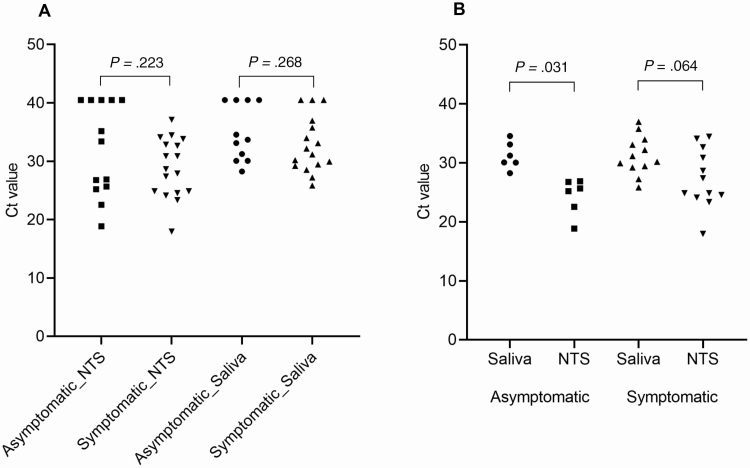
Comparison of cycle threshold values of severe acute respiratory syndrome coronavirus 2 real-time reverse-transcription polymerase chain reaction (RT-PCR) assays obtained from nasopharyngeal/throat swabs (NTSs) and saliva. *A*, Data include results of RT-PCR analysis of all available NTS and saliva samples collected from 30 participants at enrollment. *B*, Data only include results of RT-PCR analysis of paired NTS and saliva samples of 6 asymptomatic and 12 symptomatic patients who had both sample types collected at enrollment. Abbreviations: Ct, cycle threshold; NTS, nasopharyngeal/throat swab.

At enrollment, among those who were RT-PCR positive, the viral loads measured in NTS and saliva were similar in asymptomatic and symptomatic patients (Figure 3A). However, among asymptomatic patients who had both saliva and NTS samples collected, higher viral load was observed in the NTS than in saliva (*P* = .031; Figure 3B). A similar trend was observed for symptomatic cases (*P* = .064; Figure 3B).

### Quantification of Viral RNA and Duration of Viral Detection in NTS Samples

**Figure 4.**
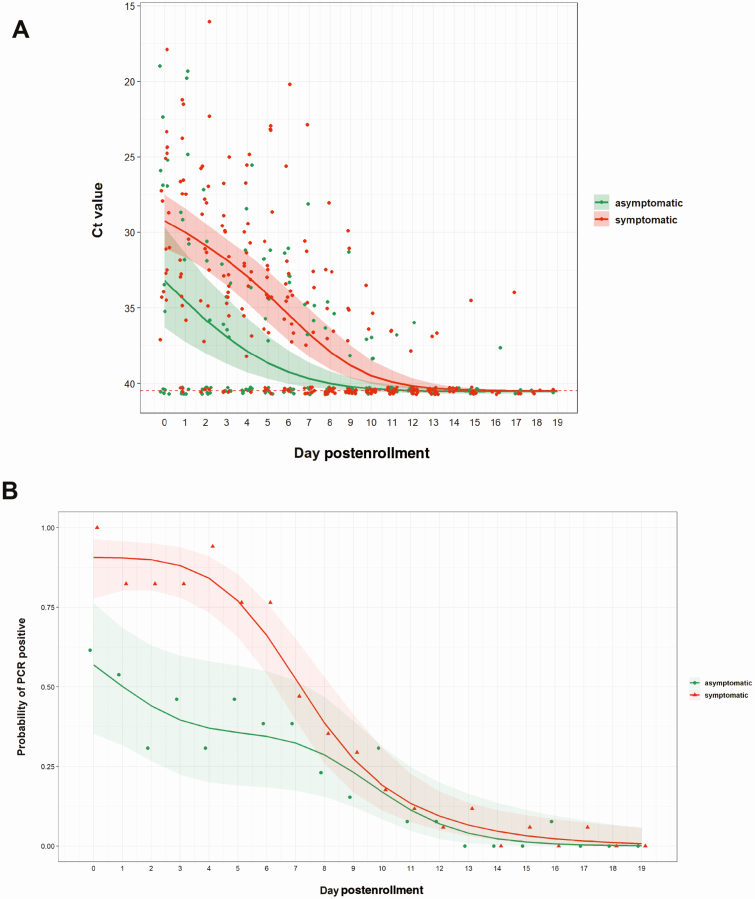
Trends in cycle threshold (Ct) values and viral detection probability in nasopharyngeal/throat swabs over the course of hospitalization. *A*, Changes of Ct values relatively reflect the level of viral load. *B*, Dynamics of viral detection probability from enrollment onward. Each dot represents 1 observed value (*A*) or the mean value (ie, frequency) per day (*B*); lines indicate mean and shades indicate 95% confidence interval. Abbreviations: Ct, cycle threshold; PCR, polymerase chain reaction.

During follow-up, Ct values differed between the 2 groups (*P* = .027 for difference over the first 19 days; Figure 4A), with asymptomatic patients having lower viral load than the symptomatic patients.

Analysis of the probability of RT-PCR positivity showed that asymptomatic participants had a lower probability of having a positive RT-PCR result (ie, a faster viral clearance) than symptomatic participants (*P* < .001 for difference over the first 19 days, Figure 4B). This difference was most pronounced during the first week of follow-up. After this period, the probability of detection quickly fell to almost zero in both groups. The majority of the positive patients were weakly positive (Ct > 32) in this period.

### Presumed Transmission From Asymptomatic Carriers

Fourteen participants were identified to have an epidemiological link with 2 community-transmission clusters occurring in HCMC during the study period. Cluster 1 had 3 patients participating in the present study. Of these 3 participants, 2 had contact with a confirmed case on 2 March who was not enrolled in this study because this patient was admitted to a different hospital. Subsequently, 1 participant developed fever, runny nose, and sore throat on 12 March 2020, suggesting an incubation period of 10 days, and tested positive for SARS-CoV-2 on 13 March 2020. The other had no fever or any signs/symptoms suggestive of infection and was positive for SARS-CoV-2 on 14 March 2020. Two days later, a colleague of these 2 cases developed mild respiratory symptoms, including runny nose and loss of sense of smell, and tested positive for SARS-CoV-2 on 17 March 2020.

**Figure 5.**
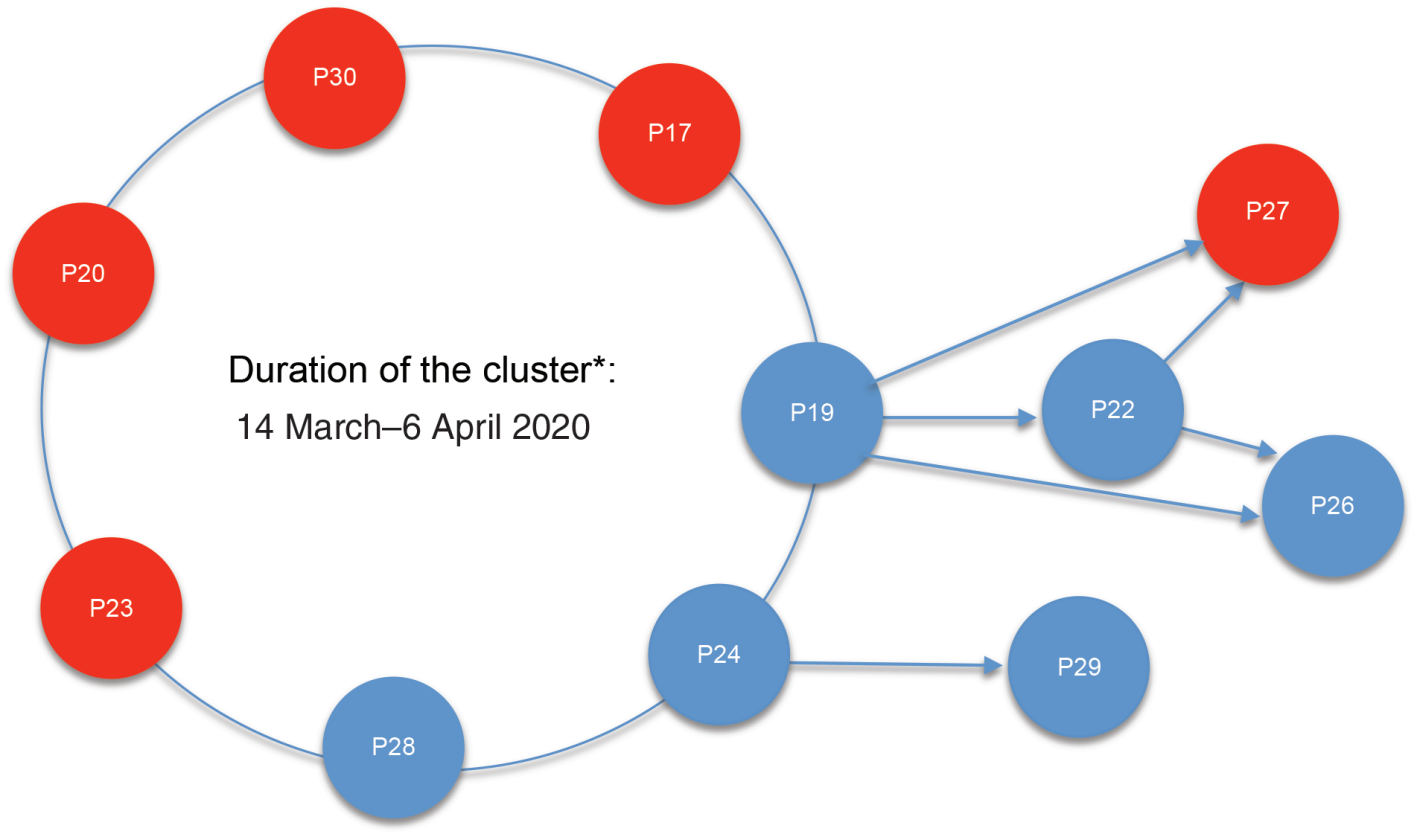
Illustration of cases with an epidemiological link with community transmission cluster 2. Red circles indicate symptomatic patients, whereas blue circles indicate asymptomatic individuals. Patients shown on the large open circle are those who first came to a local bar on 14 March 2020. Arrows indicate patients who tested positive for severe acute respiratory syndrome coronavirus 2 after having contact with individuals who attended the event on 14 March 2020. *Reflecting the period from the first contacts among individuals shown on the large open circle (14 March 2020) to the time when local health authorities completed the contact tracing activities and reverse-transcription polymerase chain reaction screening of the contacts (6 April 2020). Patient numbers correspond to the numbers presented in Supplementary Figure 2. Abbreviation: P, patient.

Cluster 2 included 11 study participants, including 6 with asymptomatic infection. Patients of this cluster were those came to a local bar on 14 March 2020, as well as individuals with whom they subsequently had contact (Figure 5). We identified a transmission chain involving an asymptomatic participant (patient 19) who was positive for SARS-CoV-2 on 23 March (Ct values: 24 for NTS and 28 for saliva). Subsequently, a contact of this case (patient 22) was positive for SARS-CoV-2 on 25 March (Ct values: 23 for NTS and 34 for saliva), although this contact did not develop symptoms. Furthermore, on 27 March, a contact of both patient 19 and 22 (patient 27) presented with cough and sore throat, with a positive NTS for SARS-COV-2. Additionally, patient 26, contact of patient 22, who was also a contact of patient 19, was confirmed with SARS-CoV-2 on 30 March, also without any symptoms. An additional transmission chain from cluster 2 was recorded between patients 24 and 29, both of whom were asymptomatic, and tested positive on 26 March and 1 April, respectively (Figure 5).

## DISCUSSION

Despite the rapid global spread of SARS-CoV-2, community transmission of SARS-CoV-2 in Vietnam remains exceptionally low [2]. Indeed, while the first reported cases date back to 23 January 2020, as of 24 April, there have been only 270 reported cases in Vietnam, including 170 imported cases and 100 cases acquired locally [2, 19]. During the same period, the number of confirmed cases worldwide increased from 582 to more than 2.7 million.

Social distancing, school closure, isolation of confirmed cases and their contacts, and airport quarantine [14, 15], coupled with RT-PCR testing for all the isolated people, have been the main measures leading to the current success of Vietnam’s COVID-19 control [14, 15]. The quarantine of large numbers of contacts has offered a unique opportunity to study the natural history of SARS-CoV-2 infection, especially in those without symptoms.

Using data from 30 patients, representing 56% of the reported cases in HCMC since the beginning of the epidemic, we provide important insights into the natural history of SARS-CoV-2 infection. We found that 43% of SARS-CoV-2–positive cases were asymptomatic, supporting previous reports [4, 7, 20, 21]. These asymptomatic carriers had comparable detection rates and viral load of SARS-CoV-2 in saliva with that of symptomatic cases. However, at enrollment and during follow-up, asymptomatic individuals had a lower probablity of having a postive RT-PCR diagnosis and had lower viral load in NTSs. Yet, despite these data suggesting faster viral clearance from the respiratory tract, we found good evidence that these asymptomatic individuals transmitted the virus to others.

SARS-CoV-2 RNA has previously been detected in saliva of COVID-19 patients [22, 23], demonstrating the utility potential of easy-to-collect saliva samples for the diagnosis of COVID-19 [24]. However, to the best of our knowledge, detection of SARS-CoV-2 in saliva of asymptomatic cases has not been previously reported. Slightly higher viral loads (lower Ct values in Figure 3) were found in NTSs than saliva, but saliva is an easier specimen to collect and may represent a better sample for mass disease–screening programs. The ease of detecting virus in the saliva is also consistent with the known high infectiousness of SARS-CoV-2 and its ready ability to spread through droplet transmission even without respiratory symptoms.

Although the viral loads at enrollment were similar between the asymptomatic and symptomatic participants, the virus appeared to be cleared faster from the respiratory tract in asymptomatic people, and faster than previously reported [13, 25]. These differences suggest that symptoms and subsequent disease severity may depend on the size of the infectious viral inoculum and/or an individual’s ability to clear the infection. However, we cannot also rule out that the time from infection to sample collection was longer in asymptomatic individuals, and/or the possibility of false-positive results of primary RT-PCR screening of the quarantined people, for example, in the case of patient 21 who became negative at enrollment (Supplementary Figure 2). Other reasons for asymptomatic infection include preexisting cross-immunity as a consequence of previous exposure to common human coronaviruses, which may enhance immunity and control of the infection in some individuals [26].

Nevertheless, despite faster viral clearance in asymptomatic individuals, we found good evidence that they were still able to transmit the infection. Two of the asymptomatic participants were the highly likely origin of at least 2, and possibly 4, further infections. Transmission from asymptomatic and especially presymptomatic individuals has been suggested previously [4–6, 8, 9] and may explain why the virus is so hard to control. The finding supports the Vietnam approach of vigorous case-finding, quarantining, and testing and suggests they are essential if the infection is to be controlled.

Both asymptomatic and symptomatic patients had the same probability of RT-PCR positivity after the first week of follow-up. This suggests that these RT-PCR–positive results may merely reflect the detection of viral RNA in respiratory samples during this later phase of the illness rather than viable viruses, in line with a recent report [13].

The strengths of our study include the inclusion of the majority of asymptomatic and symptomatic cases reported in southern Vietnam over 4 weeks, without selection bias based on symptoms or disease severity. In so doing, we were able to study prospectively the full spectrum of SARS-CoV-2 infection. Our study also has some limitations. We did not perform viral culture to demonstrate the infectiousness of SARS-CoV-2 detected by RT-PCR in saliva, although through contact history, we identified at least 2 transmission events from completely asymptomatic individuals. Additionally, we did not perform chest computed tomography scans [27], which are more sensitive than chest radiographs for the detection of lung abnormalities. Therefore, we may have underestimated the subclinical findings of SARS-CoV-2 infection. Last, none of the participants developed severe disease. However, as of 23 April 2020, only 3 severe COVID-19 cases, including one who was treated at the HTD main campus durning the study period, have been reported in HCMC and there have, as yet, been no COVID-19–related deaths in Vietnam.

To summarize, we demonstrate that a high proportion (13/30 [43%]) of quarantined people who were RT-PCR positive for SARS-CoV-2 were asymptomatic. These individuals carried SARS-CoV-2 in their respiratory tract and saliva, and were potentially contagious. They would not have been identified without the control measures as currently applied in Vietnam. Therefore, our findings emphasize the importance of contact tracing, airport quarantine, and RT-PCR screening for SARS-CoV-2 among isolated people in controlling the ongoing pandemic.

## Supplementary Data

Supplementary materials are available at *Clinical Infectious Diseases* online. Consisting of data provided by the authors to benefit the reader, the posted materials are not copyedited and are the sole responsibility of the authors, so questions or comments should be addressed to the corresponding author.

ciaa711_suppl_Supplementary_Figure_S1

ciaa711_suppl_Supplementary_Figure_S2

ciaa711_suppl_Supplementary_Material

ciaa711_suppl_Supplementary_Figure_Legends

## Notes

***Oxford University Clinical Research Unit (OUCRU) COVID-19 Research Group.*** Hospital for Tropical Diseases, Ho Chi Minh City, Vietnam: Nguyen Van Vinh Chau, Nguyen Thanh Dung, Le Manh Hung, Huynh Thi Loan, Nguyen Thanh Truong, Nguyen Thanh Phong, Dinh Nguyen Huy Man, Nguyen Van Hao, Duong Bich Thuy, Nghiem My Ngoc, Nguyen Phu Huong Lan, Pham Thi Ngoc Thoa, Tran Nguyen Phuong Thao, Tran Thi Lan Phuong, Le Thi Tam Uyen, Tran Thi Thanh Tam, Bui Thi Ton That, Huynh Kim Nhung, Ngo Tan Tai, Tran Nguyen Hoang Tu, Vo Trong Vuong, Dinh Thi Bich Ty, Le Thi Dung, Thai Lam Uyen, Nguyen Thi My Tien, Ho Thi Thu Thao, Nguyen Ngoc Thao, Huynh Ngoc Thien Vuong, Pham Ngoc Phuong Thao, Phan Minh Phuong. OUCRU, Ho Chi Minh City, Vietnam: Dong Thi Hoai Tam, Evelyne Kestelyn, Donovan Joseph, Ronald Geskus, Guy Thwaites, H. Rogier van Doorn, Ho Van Hien, Huynh Le Anh Huy, Huynh Ngan Ha, Huynh Xuan Yen, Jennifer Van Nuil, Jeremy Day, Joseph Donovan, Katrina Lawson, Lam Anh Nguyet, Lam Minh Yen, Le Nguyen Truc Nhu, Le Thanh Hoang Nhat, Le Van Tan, Sonia Lewycka Odette, Louise Thwaites, Maia Rabaa, Marc Choisy, Mary Chambers, Motiur Rahman, Ngo Thi Hoa, Nguyen Thanh Thuy Nhien, Nguyen Thi Han Ny, Nguyen Thi Kim Tuyen, Nguyen Thi Phuong Dung, Nguyen Thi Thu Hong, Nguyen Xuan Truong, Phan Nguyen Quoc Khanh, Phung Le Kim Yen, Sophie Yacoub, Thomas Kesteman, Nguyen Thuy Thuong Thuong, Tran Tan Thanh, Tran Tinh Hien, Vu Thi Ty Hang. Center for Disease Control and Prevention, Ho Chi Minh City, Vietnam: Nguyen Tri Dung, Le Hong Nga.

***Acknowledgments.*** The authors are indebted to Dr Vu Thi Ty Hang, Dr Phan Nguyen Quoc Khanh, Ms Nguyen Thanh Ngoc, Ms Le Kim Thanh, and the OUCRU Information Technology/Clinial Trial Unit/laboratory management departments, especially Mr Ho Van Hien, Mr Dang Minh Hoang, and Dr Nguyen Than Ha Quyen, for their support. The authors also

thank Professor Lin-Fa Wang and Dr Danielle Anderson of Duke–National University of Singapore Medical School for their guidance in the development of diagnostic reagents in this study; the patients for their participation in this study; and the doctors and nurses of Cu Chi Hospital, who cared for the patients and provided logistical support with the study.

***Financial support.*** This study was funded by the Wellcome Trust of Great Britain (grant numbers 106680/B/14/Z and 204904/Z/16/Z).

***Potential conflicts of interest.*** The authors: No reported conflicts of interest. All authors have submitted the ICMJE Form for Disclosure of Potential Conflicts of Interest.
