# Supplementary figures and images for: The Natural History and Transmission Potential of Asymptomatic Severe Acute Respiratory Syndrome Coronavirus 2 Infection
Source: Clin Infect Dis. 2020 Jun 4;71(10):2679–87. doi: 10.1093/cid/ciaa711 (PMC7314145; doi:10.1093/cid/ciaa711)

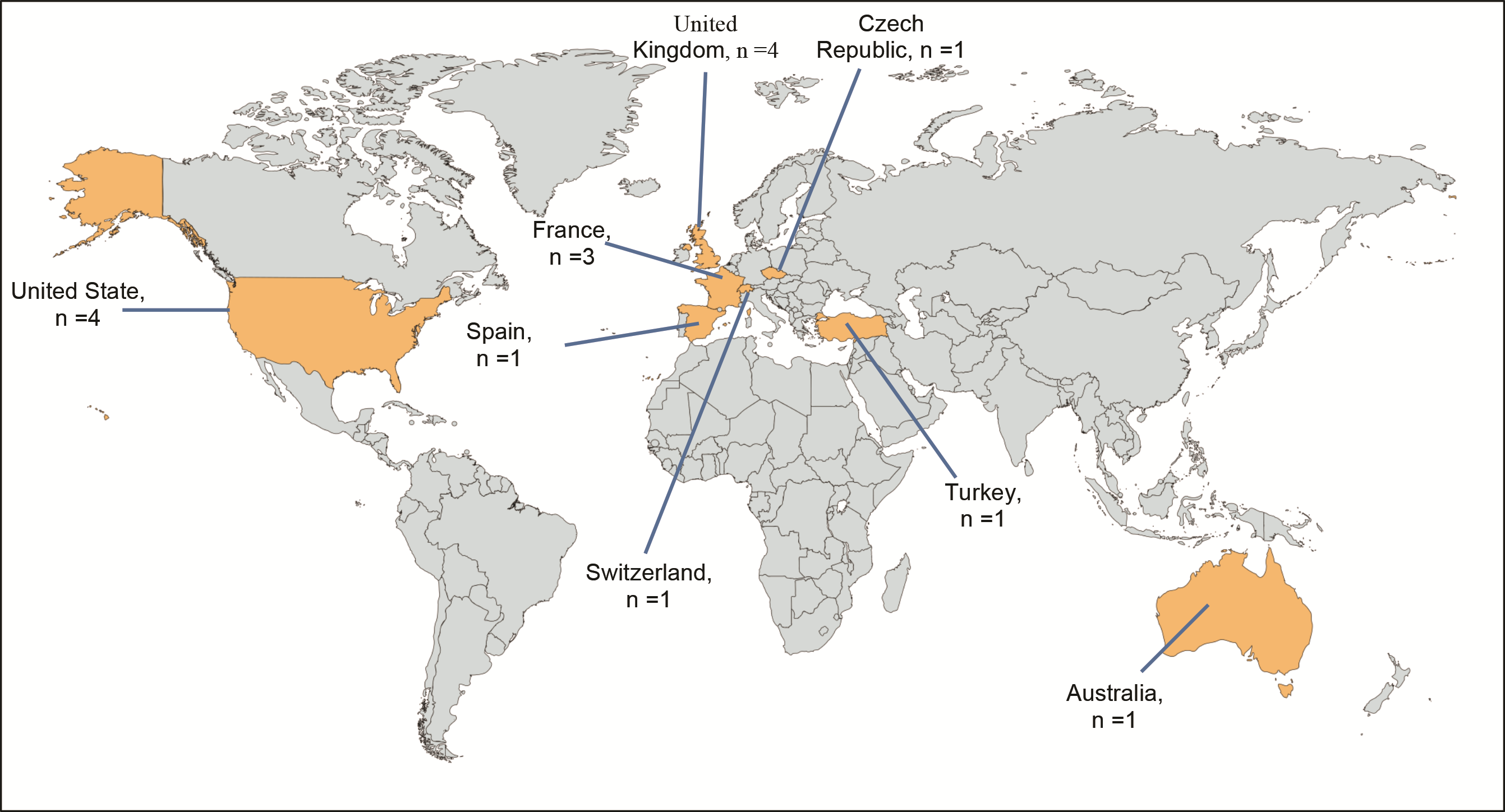

Supplement: ciaa711_suppl_Supplementary_Figure_S1 [file ciaa711_suppl_supplementary_figure_s1.png]

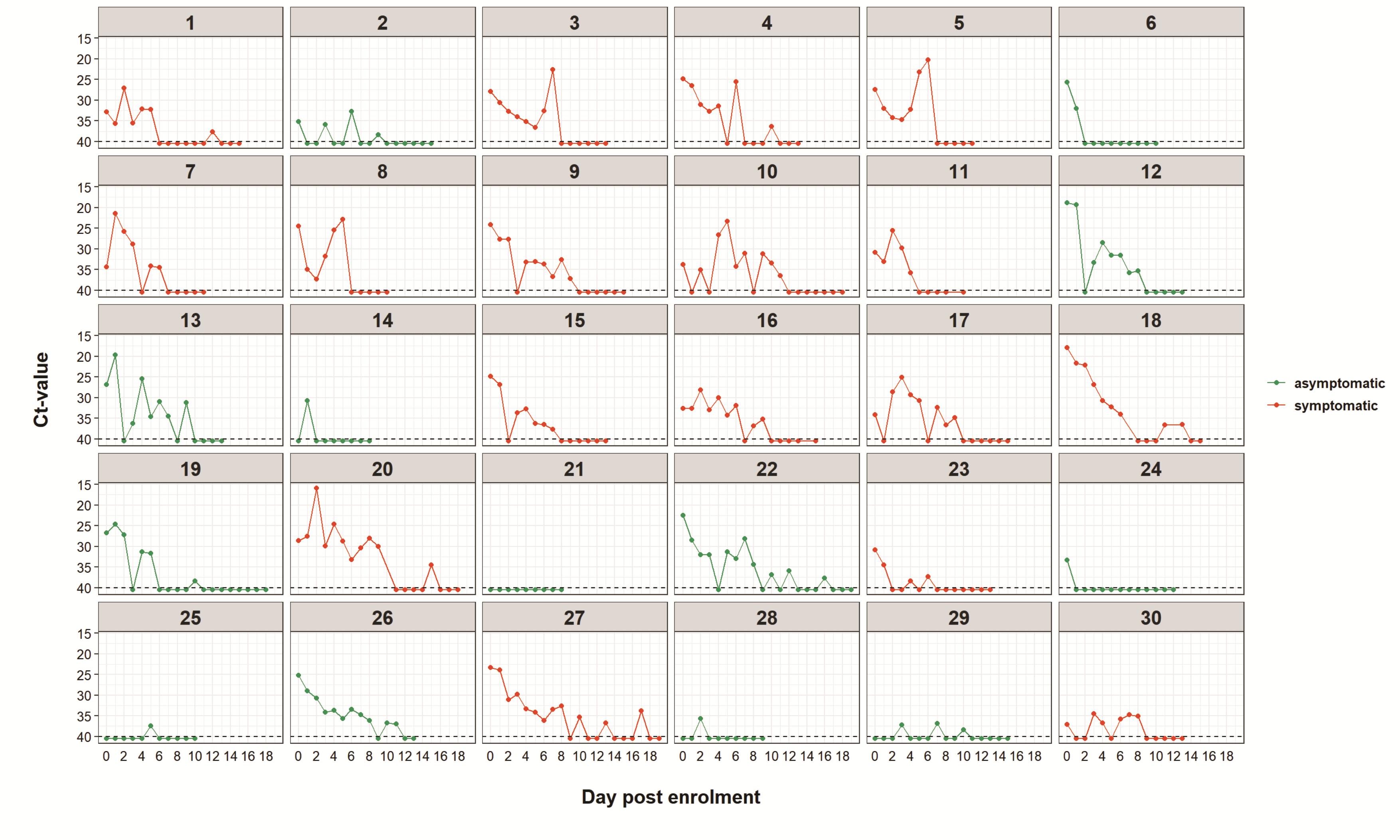

Supplement: ciaa711_suppl_Supplementary_Figure_S2 [file ciaa711_suppl_supplementary_figure_s2.png]
