# Supplementary material for: The Natural History and Transmission Potential of Asymptomatic Severe Acute Respiratory Syndrome Coronavirus 2 Infection
Source: Clin Infect Dis. 2020 Jun 4;71(10):2679–87. doi: 10.1093/cid/ciaa711 (PMC7314145; doi:10.1093/cid/ciaa711)
Supplement: ciaa711_suppl_Supplementary_Material [file ciaa711_suppl_supplementary_material.pdf]

~~~~
---
title: "The natural history of asymptomatic and symptomatic SARS-
CoV-2 infection "
authors: “Nguyen Van Vinh Chau et al.“
date: "4/23/2020"
output:
  html_document: default
---

This Rmarkdown is used to produce results and figures as used in the
paper.


```{r setup, include=FALSE}
knitr::opts_chunk$set(
        echo = TRUE,
        message = FALSE,
        warning = FALSE
)
library(tidyverse)
library(magrittr)
library(ggplot2)
theme_set(theme_bw())
library(geepack)
library(GLMMadaptive)
library(splines)
```


```{r include=FALSE}
setwd("C:/Users/nhatlth/Google Drive/COvid/")
Diagnostic_test <- as.data.frame(read_csv("Data/
Diagnostic_test_6.csv"))
Diagnostic_test_long <- Diagnostic_test %>% gather(Day, Ct, `DAY
0`:`DAY 19`, factor_key=TRUE) %>% mutate(diagnosis=ifelse(Ct=="neg",
0,1),
                            Ct_value=if_else(Ct=="neg",
40.5,as.numeric(Ct)),
                            day=as.numeric(substr(Day,4,6)),
                            pid=as.factor(ID),

Symptom=factor(Symptom,levels=c("asymptomatic","symptomatic"))) %>%
                    filter(!is.na(Ct_value)) %>%
select(pid,day,Ct_value,diagnosis,Symptom) %>%
arrange(Symptom,pid,day)
Diagnostic_test_long %<>%
mutate(Ct_value_transform=ifelse(Ct_value=="neg",0,40.5-
(as.numeric(Ct_value))))
```


```{r plot.zeroone, echo=FALSE, fig.height=9, fig.width=15}
p<-ggplot(Diagnostic_test_long, aes(day,diagnosis,colour=Symptom)) +
geom_point() + geom_line()+ facet_wrap(~pid) +
~~~~

```
scale_x_continuous(breaks=seq(0,19,by=2))+
scale_y_continuous("",breaks=c(0,1),labels=c("neg","pos"),lim=c(-0.2
,1.2)) + theme_bw()+
  theme(panel.background=element_rect(fill = 'white', colour =
'grey'),
    axis.title.x = element_text(face="bold", colour="black",
size=15, margin = margin(t = 20,r = 20,b =20, l = 20)),
    axis.title.y = element_text(face="bold", colour="black",
size=15, margin = margin(t = 20,r = 20,b = 20, l = 20)),
    axis.text.y = element_text(face="plain", colour="black",
size=10),
    axis.text.x = element_text(face="plain", colour="black",
size=10),
    axis.ticks.x=element_blank(),
    legend.text = element_text(face="bold",size = 10),
legend.title = element_blank(),         legend.key =
element_rect(colour = NA),
    strip.text=element_text(face = "bold",size=15))+
scale_colour_manual(values=c("#2ca25f","#f03b20"))
ggsave(file = "Figures/Supplementary_Figure_2.png",
    width = 15,
    height = 8, dpi=300)
p
```


Performance of PCR test result during follow-up

We use a logistic regression to compare the difference in outcome, using time as a continuous variable and modeled in a flexible way. Parameters are estimated using generalized estimated equations (GEE) in order to account for the repeated measurements within an individual. We assume an exchangeable correlation matrix.


```{r include=FALSE}
Diagnostic_test_long.imputed <- Diagnostic_test %>% gather(Day, Ct,
`DAY 0`:`DAY 19`, factor_key=TRUE) %>% mutate(
  Ct=ifelse(is.na(Ct),"neg",Ct),
  diagnosis=ifelse(Ct=="neg",0,1),
                            Ct_value=if_else(Ct=="neg",
40.5,as.numeric(Ct)),
                            day=as.numeric(substr(Day,4,6)),
                            pid=as.factor(ID),

Symptom=factor(Symptom,levels=c("asymptomatic","symptomatic"))) %>%
 select(pid,day,Ct_value,diagnosis,Symptom) %>%
arrange(Symptom,pid,day)
```



```{r include=FALSE}
```

````
fit.impute<-geeglm(diagnosis~ns(day,knots=c(3,8),Boundary.knots =
c(0,14))*Symptom,id=pid,family = binomial(link =
"logit"),data=Diagnostic_test_long.imputed,corstr="exchangeable")
fit0.impute<-geeglm(diagnosis~ns(day,knots=c(3,8),Boundary.knots =
c(0,14)),id=pid,family = binomial(link =
"logit"),data=Diagnostic_test_long.imputed,corstr="exchangeable")
p.value<-anova(fit.impute,fit0.impute)$`P(>|Chi|)`
```

* There is evidence that the two clinical groups had different
trends in positive PCR results, p.value=`r
format.pval(p.value,digits=2,eps=0.001)`

```{r message=FALSE, warning=FALSE, include=FALSE}
a<-
splines::ns(Diagnostic_test_long.imputed$day,knots=c(3,8),Boundary.k
nots = c(0,14))
knots.day<-attr(a,"knots")
day<-0:19
dat.pred<-data.frame(day =
rep(0:19,each=nlevels(Diagnostic_test_long.imputed$Symptom)),
   Symptom =factor(rep(levels(Diagnostic_test_long.imputed$Symptom),
length(day)), levels =
levels(Diagnostic_test_long.imputed$Symptom)))
mm.1<-model.matrix(~ ns(day,knots=knots.day,Boundary.knots =
c(0,14))*Symptom,data=dat.pred)
dat.pred$pred.1<-mm.1[,5:8]%*%fit.impute$coefficients[5:8]
pvar1 <- diag(mm.1[,5:8] %*%
tcrossprod(fit.impute$geese$vbeta[5:8,5:8],mm.1[,5:8]))
dat.pred$pred.1.lo <-dat.pred$pred.1-qnorm(0.975,0,1)*sqrt(pvar1)
dat.pred$pred.1.up <-dat.pred$pred.1+qnorm(0.975,0,1)*sqrt(pvar1)
dat.pred$pred<-exp(dat.pred$pred.1)
dat.pred$pred.lo <-exp(dat.pred$pred.1.lo)
dat.pred$pred.up <-exp(dat.pred$pred.1.up)
#dat.pred$Symptom<-as.factor(dat.pred$Symptom)
tab<-subset(dat.pred,Symptom=="symptomatic",select=c("day","pred",
"pred.lo", "pred.up"))
colnames(tab)<-c("day","OR","Lower.CI","Upper.CI")
tab %<>%arrange(day)
```

##### Table of comparison between two clinical groups by Day post
enrolment

```{r echo=FALSE, message=FALSE, warning=FALSE}
knitr::kable(round(tab,2))
```



```{r echo=FALSE}
a<-
splines::ns(Diagnostic_test_long.imputed$day,knots=c(3,8),Boundary.k
nots = c(0,14))
````

```
knots.day<-attr(a,"knots")
day<-0:19
dat.pred<-data.frame(day =
rep(0:19,each=nlevels(Diagnostic_test_long.imputed$Symptom)),
   Symptom =factor(rep(levels(Diagnostic_test_long.imputed$Symptom),
length(day)), levels =
levels(Diagnostic_test_long.imputed$Symptom)))
mm.1<-model.matrix(~ ns(day,knots=knots.day,Boundary.knots =
c(0,14))*Symptom,data=dat.pred)
dat.pred$pred.1<-mm.1%*%fit.impute$coefficients
pvar1 <- diag(mm.1 %*% tcrossprod(fit.impute$geese$vbeta,mm.1))
dat.pred$pred.1.lo <-dat.pred$pred.1-qnorm(0.975,0,1)*sqrt(pvar1)
dat.pred$pred.1.up <-dat.pred$pred.1+qnorm(0.975,0,1)*sqrt(pvar1)
dat.pred$pred<-plogis(dat.pred$pred.1)
dat.pred$pred.lo <-plogis(dat.pred$pred.1.lo)
dat.pred$pred.up <-plogis(dat.pred$pred.1.up)

dat.tmp<-Diagnostic_test_long.imputed %>% mutate(flag=1)%>%
group_by(day,Symptom) %>%
dplyr::summarize(PCR_pos=sum(diagnosis),no_test=sum(flag),PCR_pos_pr
op=PCR_pos/no_test) %>% ungroup() %>% as.data.frame()
dat.pred<-plyr::join_all(list(dat.pred,dat.tmp))
dat.pred$Symptom<-as.factor(dat.pred$Symptom)
```

```{r echo=FALSE, fig.height=7, fig.width=9, message=FALSE,
warning=FALSE}
p1 <- ggplot(data=dat.pred, aes(x=day,y=PCR_pos_prop,group=Symptom))
p<-
p1+geom_point(size=2,aes(shape=Symptom,colour=Symptom),position=posi
tion_dodge(width=0.5))+
  geom_line(data=dat.pred,aes(x=day,y=pred,colour=Symptom),size=1)+
  geom_ribbon(data=dat.pred,aes(ymin=pred.lo, ymax=pred.up,
x=day,fill=Symptom),alpha=0.1)+
  ylab("Probability of PCR positive")
+scale_x_continuous(breaks=0:19)+
  xlab(" Day post enrolment")+
  theme_bw()+
  theme(panel.background=element_rect(fill = 'white', colour =
'grey'),
    axis.title.x = element_text(face="bold", colour="black",
size=15, margin = margin(t = 20,r = 20,b =20, l = 20)),
    axis.title.y = element_text(face="bold", colour="black",
size=15, margin = margin(t = 20,r = 20,b = 20, l = 20)),
    axis.text.y = element_text(face="plain", colour="black",
size=10),
    axis.text.x = element_text(face="plain", colour="black",
size=10),
    axis.ticks.x=element_blank(),
    legend.text = element_text(face="bold",size = 10),
legend.title = element_blank(),         legend.key =
```

```
element_rect(colour = NA),
    strip.text=element_text(face = "bold",size=15))+
scale_colour_manual(values=c("#2ca25f","#f03b20"))
+scale_fill_manual(values=c("#2ca25f","#f03b20"))
ggsave(file = "Figures/Figure1.png",
    width = 15,
    height = 8, dpi=300)
p
```

Kinetics of Ct value assessed by two clinical groups over time

Zero-inflated mixed effects model for semi-continuous data (treat upper detection limit value as zero inflated value)

```{r eval=FALSE, include=FALSE}
hurdle.gaussian <- function () {
  stats <- make.link("identity")
  log_dens <- function (y, eta, mu_fun, phis, eta_zi) {
    sigma <- exp(phis)
    # binary indicator for y > 0
    ind <- y > 0
    # non-zero part
    eta <- as.matrix(eta)
    eta_zi <- as.matrix(eta_zi)
    out <- eta
    out[ind, ] <- plogis(eta_zi[ind, ], lower.tail = FALSE, log.p =
TRUE) +
      dnorm(x = y[ind], mean = eta[ind, ], sd = sigma, log = TRUE)
    # zero part
    out[!ind, ] <- plogis(eta_zi[!ind, ], log.p = TRUE)
    attr(out, "mu_y") <- eta
    out
  }

  simulate <- function (n, mu, phis, eta_zi) {
    y <- rnorm(n = n, mean = mu, sd = exp(phis))
    y[as.logical(rbinom(n, 1, plogis(eta_zi)))] <- 0
    y
  }
  structure(list(family = "two-part gaussian", link = stats$name,
                 linkfun = stats$linkfun, linkinv = stats$linkinv,
log_dens = log_dens,
                 simulate = simulate),
            class = "family")
}
dat<-Diagnostic_test_long
fit2.zero <- mixed_model(Ct_value_transform ~ Symptom*day, random =
~ day | pid, zi_random = ~ 1 | pid, data = dat,family =
hurdle.gaussian(), n_phis = 1,zi_fixed = ~ Symptom*day)
fit1.zero <- mixed_model(Ct_value_transform ~ day, random = ~ day |
pid, zi_random = ~ 1 | pid, data = dat,family = hurdle.gaussian(),
n_phis = 1,zi_fixed = ~ Symptom*day)
```

````
fit1.zero.intercept <- mixed_model(Ct_value_transform ~ Symptom+day,
random = ~ day | pid, zi_random = ~ 1 | pid, data = dat,family =
hurdle.gaussian(), n_phis = 1,zi_fixed = ~ Symptom*day)
save(fit1.zero,file="fit1_zero.Rdata")
save(fit1.zero.intercept,file="fit1_zero_intercept.Rdata")
save(fit2.zero,file="fit2_zero.Rdata")
```


```{r echo=FALSE}
load(file="fit2_zero.Rdata")
summary(fit2.zero)
```


```{r echo=FALSE, fig.height=7, fig.width=9, message=FALSE,
warning=FALSE}
dat<-effectPlotData(fit2.zero,newdata = Diagnostic_test_long)
dat$pred<-40.5-dat$pred
dat$pred.lo<-40.5-dat$upp
dat$pred.up<-40.5-dat$low

p1 <- ggplot(data=dat, aes(day,pred,group=Symptom))
p<-p1+geom_line(size=1,aes(colour=Symptom))+
  geom_ribbon(aes(ymin=pred.lo, ymax=pred.up, x=day,fill=Symptom),
alpha = 0.3)+geom_jitter(data=Diagnostic_test_long,
aes(day,Ct_value,colour=Symptom),width = 0.25,height = 0.25) +
  ylab("Ct value ")+scale_x_continuous(breaks=0:19)+
  xlab(" Day post enrolment")+scale_y_reverse()+
  theme_bw()+
  theme(panel.background=element_rect(fill = 'white', colour =
'grey'),
    axis.title.x = element_text(face="bold", colour="black",
size=15, margin = margin(t = 20,r = 20,b =20, l = 20)),
    axis.title.y = element_text(face="bold", colour="black",
size=15, margin = margin(t = 20,r = 20,b = 20, l = 20)),
    axis.text.y = element_text(face="plain", colour="black",
size=10),
    axis.text.x = element_text(face="plain", colour="black",
size=10),
    axis.ticks.x=element_blank(),
    legend.text = element_text(face="bold",size = 10),
legend.title = element_blank(),         legend.key =
element_rect(colour = NA),
    strip.text=element_text(face = "bold",size=15))
+geom_hline(yintercept=40.5,color='red',linetype = "dashed")+
scale_colour_manual(values=c("#2ca25f","#f03b20"))
+scale_fill_manual(values=c("#2ca25f","#f03b20"))
ggsave(file = "Figures/Figure4A.png",
    width = 15,
    height = 8, dpi=300)
p

```
````
````

````
```{r include=FALSE}
load(file="fit2_zero.Rdata")
load(file="fit1_zero.Rdata")
```


If we used loglik to compare two nested models: one allows for
diffrent time trend between two clinical groups and one with only
one time trend then there is significant difference between two time
trends with p.value=`r format.pval(anova(fit1.zero, fit2.zero)
$p.value,digits=2,eps=0.001)`.

Dynamics of Ct-value among those PCR+
```{r}
dat<-subset(Diagnostic_test_long,Ct_value_transform>0)
library(lme4)
fit3<- lmer(Ct_value_transform ~
Symptom*ns(day,knots=c(3,6),Boundary.knots = c(0,10))+(day| pid),
data = dat)
fit4<- lmer(Ct_value_transform ~ ns(day,knots=c(3,6),Boundary.knots
= c(0,10))+(day| pid), data = dat)
a<-anova(fit3,fit4)$`Pr(>Chisq)`
```

Test for the difference between two groups: p.value=`r
format.pval(a[2],digits=2,eps=0.001)`

```{r echo=FALSE, fig.height=7, fig.width=9, message=FALSE,
warning=FALSE}
mm.1<-model.matrix(~ ~ Symptom*ns(day,knots=c(3,6),Boundary.knots =
c(0,10)),data=dat)
dat$pred.1<-mm.1%*%fixef(fit3)
pvar1 <- diag(mm.1 %*% tcrossprod(summary(fit3)$vcov,mm.1))
dat$pred.1.lo <-dat$pred.1-qnorm(0.975,0,1)*sqrt(pvar1)
dat$pred.1.up <-dat$pred.1+qnorm(0.975,0,1)*sqrt(pvar1)
dat$pred<-40.5-dat$pred.1
dat$pred.lo<-40.5-dat$pred.1.up
dat$pred.up<-40.5-dat$pred.1.lo


p1 <- ggplot(data=dat, aes(day,pred,group=Symptom))
p<-p1+geom_line(size=1,aes(colour=Symptom))+
  geom_ribbon(aes(ymin=pred.lo, ymax=pred.up, x=day,fill=Symptom),
alpha = 0.3)+geom_jitter(data=dat,
aes(day,Ct_value,colour=Symptom),width = 0.25,height = 0.25) +
  ylab("Ct value ")+scale_x_continuous(breaks=0:19)+
  xlab(" Day post enrolment")+scale_y_reverse()+
````
````

```
  theme_bw()+
  theme(panel.background=element_rect(fill = 'white', colour =
'grey'),
    axis.title.x = element_text(face="bold", colour="black",
size=15, margin = margin(t = 20,r = 20,b =20, l = 20)),
    axis.title.y = element_text(face="bold", colour="black",
size=15, margin = margin(t = 20,r = 20,b = 20, l = 20)),
    axis.text.y = element_text(face="plain", colour="black",
size=10),
    axis.text.x = element_text(face="plain", colour="black",
size=10),
    axis.ticks.x=element_blank(),
    legend.text = element_text(face="bold",size = 10),
legend.title = element_blank(),         legend.key =
element_rect(colour = NA),
    strip.text=element_text(face = "bold",size=15))
+geom_hline(yintercept=40.5,color='red',linetype = "dashed")+
scale_colour_manual(values=c("#2ca25f","#f03b20"))
+scale_fill_manual(values=c("#2ca25f","#f03b20"))
ggsave(file = "Figures/SubFigure3.png",
    width = 15,
    height = 8, dpi=300)
p
```
