# Supplementary material for: The Natural History and Transmission Potential of Asymptomatic Severe Acute Respiratory Syndrome Coronavirus 2 Infection
Source: Clin Infect Dis. 2020 Jun 4;71(10):2679–87. doi: 10.1093/cid/ciaa711 (PMC7314145; doi:10.1093/cid/ciaa711)
Supplement: ciaa711_suppl_Supplementary_Figure_Legends [file ciaa711_suppl_supplementary_figure_legends.docx]

**Supplementary Figure 1:** Map showing countries where the imported cases stayed before travelling to Vietnam

**Note to supplementary Figure 1**: Maps were obtained from  <https://mapchart.net/>

**Supplementary Figure 2:** Individuals data on Ct values of SARS-CoV-2 real-time RT-PCR obtained from analysis of nasopharyngeal throat swabs collected at enrollment and during follow-up

**Note to Supplementary Figure 2**: Horizontal dash lines indicate the assay cut off.
